# Targeted elimination of mutant mitochondrial DNA in MELAS-iPSCs by mitoTALENs

**DOI:** 10.1007/s13238-017-0499-y

**Published:** 2018-01-09

**Authors:** Yi Yang, Han Wu, Xiangjin Kang, Yanhui Liang, Ting Lan, Tianjie Li, Tao Tan, Jiangyun Peng, Quanjun Zhang, Geng An, Yali Liu, Qian Yu, Zhenglai Ma, Ying Lian, Boon Seng Soh, Qingfeng Chen, Ping Liu, Yaoyong Chen, Xiaofang Sun, Rong Li, Xiumei Zhen, Ping Liu, Yang Yu, Xiaoping Li, Yong Fan

**Affiliations:** 1 Key Laboratory for Major Obstetric Diseases of Guangdong Province, Key Laboratory of Reproduction and Genetics of Guangdong Higher Education Institutes, The Third Affiliated Hospital of Guangzhou Medical University, Guangzhou, 510150 China; 2 Key Laboratory of Regenerative Biology of the Chinese Academy of Sciences and Guangdong Provincial Key Laboratory of Stem Cells and Regenerative Medicine, South China Institute for Stem Cell Biology and Regenerative Medicine, Guangzhou Institutes of Biomedicine and Health, Chinese Academy of Sciences, Guangzhou, 510530 China; 3 Center of Reproductive Medicine, Department of Obstetrics and Gynecology, Peking University Third Hospital, Beijing, 100191 China; 4 Yunnan Key Laboratory of Primate Biomedical Research, Institute of Primate Translational Medicine, Kunming University of Science and Technology, Kunming, 650500 China; 5 Disease Modeling and Therapeutics Laboratory, A*STAR Institute of Molecular and Cell Biology, 61 Biopolis Drive Proteos, Singapore, 138673 Singapore; 6 Department of Biological Sciences, National University of Singapore, Singapore, 117543 Singapore

**Keywords:** mitochondria, iPSCs, TALEN, MELAS

## Abstract

Mitochondrial diseases are maternally inherited heterogeneous disorders that are primarily caused by mitochondrial DNA (mtDNA) mutations. Depending on the ratio of mutant to wild-type mtDNA, known as heteroplasmy, mitochondrial defects can result in a wide spectrum of clinical manifestations. Mitochondria-targeted endonucleases provide an alternative avenue for treating mitochondrial disorders via targeted destruction of the mutant mtDNA and induction of heteroplasmic shifting. Here, we generated mitochondrial disease patient-specific induced pluripotent stem cells (MiPSCs) that harbored a high proportion of m.3243A>G mtDNA mutations and caused mitochondrial encephalomyopathy and stroke-like episodes (MELAS). We engineered mitochondrial-targeted transcription activator-like effector nucleases (mitoTALENs) and successfully eliminated the m.3243A>G mutation in MiPSCs. Off-target mutagenesis was not detected in the targeted MiPSC clones. Utilizing a dual fluorescence iPSC reporter cell line expressing a 3243G mutant mtDNA sequence in the nuclear genome, mitoTALENs displayed a significantly limited ability to target the nuclear genome compared with nuclear-localized TALENs. Moreover, genetically rescued MiPSCs displayed normal mitochondrial respiration and energy production. Moreover, neuronal progenitor cells differentiated from the rescued MiPSCs also demonstrated normal metabolic profiles. Furthermore, we successfully achieved reduction in the human m.3243A>G mtDNA mutation in porcine oocytes via injection of mitoTALEN mRNA. Our study shows the great potential for using mitoTALENs for specific targeting of mutant mtDNA both in iPSCs and mammalian oocytes, which not only provides a new avenue for studying mitochondrial biology and disease but also suggests a potential therapeutic approach for the treatment of mitochondrial disease, as well as the prevention of germline transmission of mutant mtDNA.

**Electronic supplementary material:**

The online version of this article (10.1007/s13238-017-0499-y) contains supplementary material, which is available to authorized users.

## Introduction

Mitochondria, the so-called “powerhouses” of cells, are double-membrane cellular organelles that are found in almost all eukaryotic cells. They play major roles in multiple cellular processes, including energy production, calcium homoeostasis, cellular signaling, and apoptosis (Chan, 2006). Unlike most other organelles, mitochondria contain their own circular genomic DNA (mtDNA) and reproduce independently of cells. mtDNA encodes a host of enzymes, as well as ribosomes for protein synthesis, which critically catalyze many metabolic steps of cellular respiration (Anderson et al., 1981). In contrast to nuclear DNA, mtDNA is exclusively transmitted through maternal inheritance (Taylor and Turnbull, 2005). In contrast to cells, mitochondria only possess a base-excision repair pathway to repair oxidative DNA damage; a double-stranded break (DSB) repair pathway has not been identified in mitochondria. Mutations in mtDNA occur at a higher rate than in nuclear DNA (Moretton et al., 2017), and mutations in mtDNA cause a group of maternally inherited genetic disorders termed mitochondrial diseases, which affect 1 in 5,000 live births and cause significant morbidity and mortality (Haas et al., 2007). In most mitochondrial diseases, mutant mtDNA co-exists with wild-type mtDNA, resulting in a situation of mtDNA heteroplasmy in which the residual wild-type mtDNA can partially compensate for the mutated mtDNA, averting a complete bioenergetic crisis (Wallace, 2013). However, when the percentage of mutant mtDNA exceeds a threshold in the range of 60%–95%, depending on the severity of the mutation, pathogenic mtDNA mutations can lead to various clinical manifestations (Russell and Turnbull, 2014).

To date, the only therapeutic option for preventing the transmission of mtDNA mutations is transferring embryos below the threshold of clinical expression based on preimplantation genetic diagnosis (PGD) (Brown et al., 2006). However, PGD can only partially reduce, not completely eliminate, the risk of transmitting mutant mtDNA. Moreover, embryo viability may be affected by the PGD procedure, which includes micromanipulation of the blastomeres (Reddy et al., 2015). Recently, mitochondrial replacement therapy (MRT) has emerged as a strategy for preventing the inheritance of mtDNA by using enucleated donor oocytes to replace defective maternal mitochondria (Wang et al., 2014; Wu et al., 2017). However, these techniques require combining genetic material from different individuals, which has raised medical, safety, and ethical concerns (Vogel, 2014). In addition, a recent study also showed that embryos reconstructed by MRT still show levels of heteroplasmy, which might impact the stability of the mtDNA genotype and compromise the efficacy of mitochondrial replacement (Yamada et al., 2016).

By contrast, the use of mitochondria-targeted nucleases has shown promise regarding their ability to target mutant mtDNA. Previous studies have demonstrated that mitochondria-targeted restriction endonucleases can be used to eliminate mutant mtDNA and to alter mtDNA heteroplasmy in living cells (Alexeyev et al., 2008) and mice (Reddy et al., 2015; Bacman et al., 2010). Moreover, customized endonucleases, such as transcription activator-like effector nucleases (TALENs) (Bacman et al., 2013) and zinc finger nucleases (ZFNs) (Minczuk et al., 2006), targeted to mitochondria have been utilized for the specific elimination of mitochondrial genomes carrying mutations responsible for mitochondrial diseases. In addition, these customized nucleases allow the development of specific nucleases against essentially any DNA sequence, overcoming the main limitation of restriction endonucleases, which typically have strict and limited DNA sequence recognition capacities (Bacman et al., 2013). However, these approaches have only been demonstrated in human tumor-derived cell lines or mouse models harboring mtDNA mutations. Characterizing the effects of mitochondria-targeted nucleases in patient-derived primary cells, especially pluripotent cells, is critical for our understanding of the practical application of mitochondria-targeted nucleases for the treatment of mitochondrial diseases and the prevention of germline transmission of mutant mtDNA.

To address this issue, we generated induced pluripotent stem cells (iPSCs) from a patient with mitochondrial encephalomyopathy and stroke-like episodes (MELAS) (Goto et al., 1990) caused by an m.3243A>G heteroplasmic mutation in tRNA^Leu^ (MT-TL1). Multiple iPSC lines demonstrating high m.3243A>G heteroplasmy were generated. We then engineered transcription activator-like effector nucleases (TALENs) to specifically localize to the mitochondria and cleave pathogenic m.3243A>G mutant mtDNA. Transient expression of mitochondria-targeted TALENs (mitoTALENs) achieved a remarkable reduction in mutant mtDNA levels in patient-specific iPSCs. Importantly, genetically rescued patient-specific iPSCs and differentiated NPCs displayed normal metabolic functions, and no off-target effects on either mitochondrial or nuclear genomes were detected with the mitoTALENs. In addition, we also successfully achieved a reduction in the levels of human m.3243A>G mtDNA mutation in porcine oocytes using mitoTALENs. This study demonstrated the capacity of mitoTALENs to specifically target mtDNA mutations and induce metabolic heteroplasmic shifting in patient-derived iPSCs.

## Results

### iPSCs derived from a patient with MELAS

**Figure 1:**
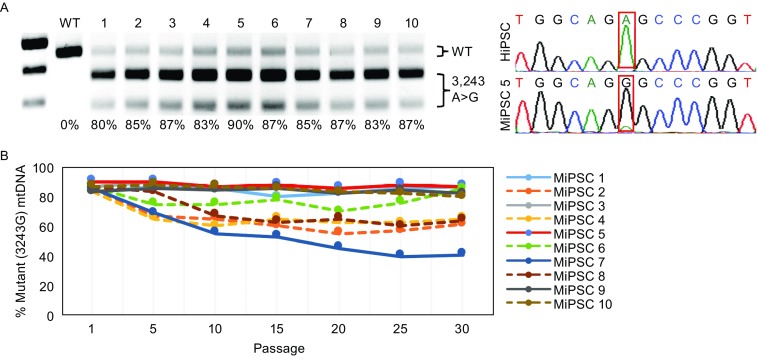
**Derivation and characterization of MELAS patient-specific iPSCs harboring a mtDNA 3243A>G mutation**. (A) MELAS patient-specific iPSC (MiPSC) lines were established that harbored high 3243A>G heteroplasmy levels (>80%). The mtDNA 3243A>G mutation rate was determined by RFLP analysis and Sanger sequencing. An iPSC line derived from healthy human fibroblasts (HiPSC) was used as the control. (B) The MiPSC lines maintained high 3243A>G heteroplasmy levels after long-term culture. The 3243A>G heteroplasmy levels were assessed in MiPSC lines every 5 passages from passage 1 to passage 30

Primary fibroblasts carrying 90% 3243A>G mutant mtDNA were derived from skin biopsy samples of a MELAS patient. MiPSCs were generated by reprogramming fibroblast cells under transgene-free, serum-free, and feeder-free conditions. The mutation rate of mtDNA 3243A>G in the patient-derived MiPSCs clones was measured by PCR restriction fragment length polymorphism (RFLP) (Zhang et al., 2015) and Sanger sequencing. As previously reported, the reprogramming of somatic cells from mitochondrial disease patients resulted in varying mutation rates in the iPSCs (Fig. S1A) (Folmes et al., 2013; Kodaira et al., 2015). Nevertheless, primary MiPSCs lines harboring 3243A>G heteroplasmy levels >80% were established (Fig. 1A). While the mutation rates continued to vary during long-term culture, most MiPSC lines maintained stable heteroplasmy levels (Fig. 1B). The observed fluctuations in heteroplasmy levels could be due to the different reprogramming states of the cell lines, i.e., highly pluripotent iPSCs predominantly produce energy by anaerobic glycolysis and carry a high proportion of mutations, even above the pathogenic threshold level. The MiPSC5 cell line was selected for the remainder of the study, as the heteroplasmy level was maintained stably in each passage.

To characterize the MELAS-iPSCs, we analyzed their pluripotency and differentiation capacity at passage 30. The expression levels of the pluripotent markers OCT4, SOX2, TRA1-60, SSEA4 and NANOG (Fig. S1B) were measured by immunofluorescence staining. We further assessed the differentiation ability of the reprogrammed MiPSCs based on the formation of teratomas *in vivo*. Histological examination revealed that the teratomas consisted of three embryonic germ layers, including intestine-like tissues (endoderm), cartilage (mesoderm), and neuron-like tissues (ectoderm) (Fig. S1C). Further analysis showed that the MiPSCs contained normal chromosomes (Fig. S1C).

### Engineered mitoTALENs specifically target the mitochondrial genome

**Figure 2:**
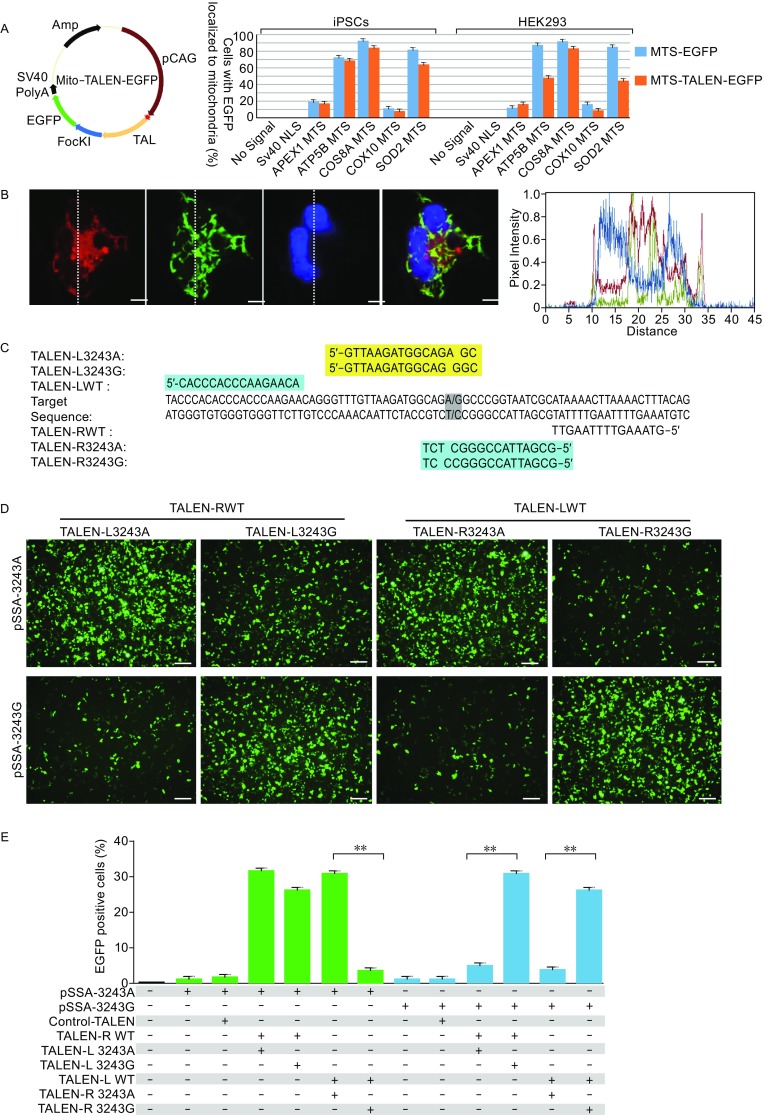
**Engineering of mitoTALENs that specifically target 3243G mutant mtDNA**. (A) The specificity of mitochondrial localization of EGFP and the TALEN-EGFP fusion proteins mediated by MTS derived from different nuclear genes. Left, schematic drawing of the engineered TALEN-EGFP polypeptide monomer containing an MTS in the N-terminus (MLS). The specificity and efficiency of MTS targeting to the mitochondria were analyzed based on the percentage of the EGFP and MitoTracker co-localizing cells in all of the transfected iPSCs and HEK293 cells, as shown in Fig. S1B and S1C. (B) Mitochondrial localization of mitoTALEN monomers mediated by COX8A-MTS in iPSCs 24 h after transfection. Mitochondria were visualized by MitoTracker Red. Scale bar = 10 µmol/L. Co-localization was visualized by the overlapping peaks of the relative fluorescence intensity (y-axis) on lines that passed through areas with marker signal. The position of the lines is indicated on the images, with lines running from top to bottom. (C) TALENs designed for targeting the mtDNA 3243 locus. (D and E) A single-strand annealing (SSA) assay to determine the specific targeting of the 3243G mtDNA mutation by TALENs. The mutated EGFP coding sequence was divided into two segments, which were separated by a stop codon and targeting sequence. Both segments contained an identical homology region. Once double-strand breaks were introduced into the target site by TALENs, the mutated EGFP coding sequence was repaired by annealing the two homologous sequences. Expression of EGFP was detected with a confocal microscope using appropriate filters after 48 h (D), (scale bars 50 μm), and the proportion of the EGFP-positive cells was measured by flow cytometry (E) (*n* = 3, error bars represent ±SEM; **P < 0.05)

As the mitochondrial genome encodes only approximately 1% of the total mitochondrial proteome, and the remaining 99% of the mitochondrial proteins encoded by the nuclear genome, mitochondrial proteins synthesized in the cytosol require mitochondrial targeting signals (MTS) to facilitate their import into the mitochondria (Lionaki et al., 2016). Using MTS, gene therapies for mitochondrial disorders have been developed by delivering functional proteins and endonucleases to mitochondria with endogenous MTS (Farrar et al., 2013). To engineer specific mitochondrial-targeted TALENs (MitoTALENs), we first assessed the mitochondrial localization of exogenous proteins driven by different endogenous MTSs using fluorescence colocalization analysis. MTS derived from nuclear genes (APEX1 (Li et al., 2010), ATP5B (Reddy et al., 2015), COX8A (Bacman et al., 2013), COX10 (Smith et al., 2012), and SOD2 (Bacman et al., 2013)) were used to construct mitochondria-targeted EGFP (mito-EGFP) and TALEN-EGFP (mitoTALEN-EGFP) by fusing the MTS at the N-terminus (Figs. 2A, S2A, and S2B). The mito-EGFP and mitoTALEN-EGFP expression vectors were transiently transfected into human iPSCs and HEK293 cells by electroporation. EGFP and TALEN-EGFP with a SV40-nuclear localization signal (NLS) and without any localization signal (controls) were also generated. Strong GFP expression was detected in all transfected cells 24 h post-transfection. The cells were fixed with 4% PFA and stained with a mitochondrial marker (Mitotracker) and a nuclear marker (Hoechst 33342). The specificity of the selected MTS targeting of mitochondria was determined by analyzing the co-localization of green fluorescence with Mitotracker and not Hoechst 33342 (Fig. S2B and S2C). Among the five selected MTS, three were derived from ATP5B, COX8A and SOD2 and showed higher specificity towards targeting the mitochondria in both HEK293 and in hiPS cells (Fig. 2A). COX8A-MTS was selected for construction of the mitochondrial-targeted TALENs, as it showed the highest capacity for mitochondrial localization (Fig. 2A and 2B).

Two pairs of mitoTALENs were designed to target the 3243G mtDNA mutation (Fig. 2C). The principle for the design of mitoTALENs is that each monomer for targeting the mitochondrial DNA contains 14.5–16.5 repeats: one monomer can bind the mutated sequence, in which the mutated site is adjacent to the 3′ end of the targeting sequence, and the other monomer is targeted to the wild-type sequence with a 14–17 bp spacer length, dictating the specific cleavage of the mutant mtDNA upon dimerization of the FokI nuclease. To evaluate the specificity and cleavage activity of the mitoTALENs, we generated a single-strand annealing (SSA) assay vector harboring either the mutant 3243G or the wild-type 3243A mtDNA sequence. We also generated mitoTALENs targeting the wild-type sequence as controls (Fig. 2C). After co-transfection of mitoTALEN pairs with the SSA assay vector into HEK293 cells, the percentage of restored EGFP-positive cells was determined via fluorescence microscopy and flow cytometry. We found that the mitoTALEN pairs TALEN-LWT/TALEN-R3243, which targeted to the antisense strand mtDNA 3,243 locus, showed significantly different cleavage activities on mutated and wild-type sequences. However, the mitoTALEN pairs TALEN-L3243/TALEN-RWT had similar efficiencies in the binding and cleavage of both mutant and wild-type target sequences (Fig. 2D and 2E). Therefore, the subsequent experiments were performed using the mitoTALEN pairs TALEN-LWT/TALEN-R324.

### Specific targeting of mutant mtDNA in patient-derived iPSCs by mitoTALENs

**Figure 3:**
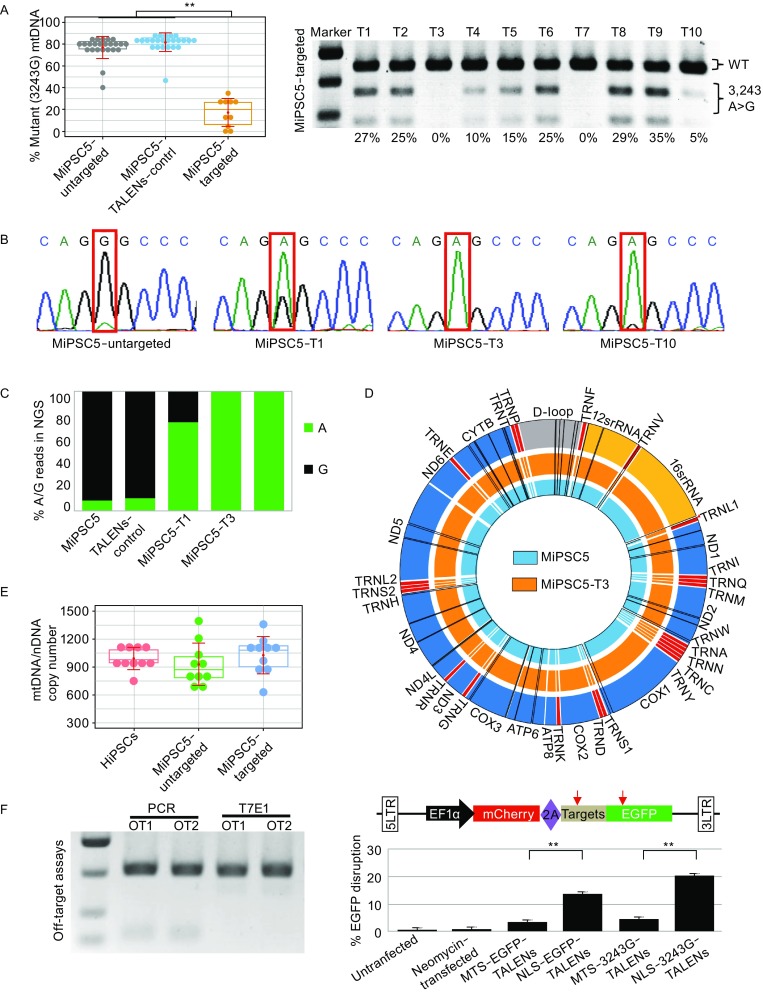
**MitoTALENs specifically target and eliminate mutant mtDNA in MELAS-iPSCs**. (A) RFLP analysis and quantification of mtDNA 3243A>G heteroplasmy in MELAS-iPSCs and subclones targeted by mitoTALENs (MiPSC5-untargeted *n* = 24; MiPSC5-TALEN-control *n* = 24, MiPSC5-targeted *n* = 10, error bars represent ±SEM; **P < 0.05). (B) Sanger sequencing to determine the mtDNA 3243A>G heteroplasmy in targeted MiPSC clones. Chromatographs show mtDNA genotyping at the 3,243 position (red box) in representative targeted MELAS-iPSC subclones. (C) Percentages of A and G reads at the mtDNA 3,243 position of the MELAS-iPSCs and targeted subclones were quantified using next-generation sequence analysis. (D) Single-nucleotide variations (SNVs) in iPSCs via exome sequencing. The black bars represent individual SNVs. Compared with untargeted b-thal iPSCs, iPSC-C2 had 12 SNVs, and the remaining corrected colonies had 21 SNVs. (E) mtDNA/nuclearDNA (nDNA) ratio in wild-type hiPSCs, MELAS-iPSCs and targeted clones (*n* = 10, error bars represent ±SEM). (F) T7E1 assays assessed mutagenesis at 2 predicted off-target sites; PCR products were used as a control. (G) Dual-fluorescence reporter-based assay assessing the nuclear targeting of mitoTALENs (*n* = 3, error bars represent ±SEM; **P < 0.05)

Encouraged by the high specificity and cleavage activity of the mitoTALENs targeting the SSA assay vectors, we next tested the mitoTALENs TALEN-LWT + TALEN-R3243G in MiPSC5 sub-lines harboring approximately 90% mutant m.3243A>G mtDNA. The mitoTALENs, together with an EGFP-expression plasmid, were electroporated into the iPSCs. EGFP-positive cells were sorted by FACS 48 h after electroporation and propagated into sub-clones for heteroplasmy testing. The EGFP-expression plasmid and a pair of untargeted mitoTALENs (TALEN-control) were also transfected as a control. The 3243A>G heteroplasmy levels were assessed using RFLP after the sub-clones were expanded for one week. Compared with the controls, 3243A>G MitoTALENs significantly reduced the percentage of mutant mtDNA in all of the sub-clones derived from the MELAS-iPSCs (Figs. 3A, 3B, and S3A). Interestingly, the mutant mtDNA was completely undetectable in two 3243G mitoTALEN-transfected MiPSC subclones (MiPSC5-T3 and MiPSC5-T7) by RFLP and Sanger sequencing. We then performed sequencing of the entire mtDNA genome using the Illumina MiSeq platform and quantitated the 3243A>G mutation ratio in MiPSC5 and the sub-clones (Fig. 3C). 92% and 89% 3243A>G heteroplasmy was detected in the MiPSC5 and TALEN-control cells, respectively, 27% 3243A>G heteroplasmy was detected in the targeted MiPSC5-T1 sub-clone, and the targeted MiPSC5-T3 and MiPSC5-T7 cells were homoplasmic for the wild-type allele. In addition to the pathogenic 3243A>G mutations, two heteroplasmic variants in the 16S rRNA gene and in MT-ATP6 were different in the MiPSC5 and targeted sub-clones (Fig. 3D, Table S1). In addition, in all of those clones, we also detected another 7 single nucleotide polymorphisms (SNPs) in the D-loop region, 2 in the 12S rRNA gene, 2 in the 16S rRNA gene, 1 in the tRNA-R gene, and 23 in protein genes (Fig. 3D, Table S1). Clinical symptoms associated with these variants have not been reported. The mtDNA copy number was determined by qPCR as previously reported (Rooney et al., 2015); compared with WT hiPSC and untargeted MiPSCs, no significant changes were detected in targeted MiPSC clones (Fig. 3E). The pluripotency and differentiation capacity of the targeted sub-clones were also assessed by immunofluorescence staining of pluripotency markers and by teratoma formation. Cytogenetic G-banding analysis revealed that targeted sub-clones retained normal diploid karyotypes with no detectable numerical or structural chromosomal abnormalities (Fig. S3B and S3C). We also performed fingerprinting by short tandem repeat analysis (STR) and confirmed that all of the MiPSC5 and targeted sub-clones were derived from the same patient (Fig. S3D).

To assess potential off-target effects on the nuclear genome, we first computationally predicted off-target sites in the human genome potentially created by the mitoTALENs. Only two potential off-target sites (Table S2) were found in the human genome with fewer than four mismatches/gaps in the spacer region. A T7 endonuclease I (T7EI) assay and Sanger sequencing were performed to detect the off-target cleavage of the mitoTALENs. No mutations were found in the potential off-target sites in any of the targeted sub-clones (Fig. 3F). Additionally, we did not find a target site for the mtDNA 3243G mitoTALENs in NMTRL-TAA3-1, the nuclear-encoded mitochondrial transfer RNA-Leu gene.

To further assess the off-target effects of mitoTALENs on nuclear genomes, we generated a dual-fluorescence reporter lentivirus vector, pEF1α-mCherry-EGFP, which harbored the mtDNA 3243G mitoTALEN targeting sequence between mCherry and the EGFP cassette (Fig. 3G). Approximately 27 h after iPSCs were infected with the dual-fluorescence reporter lentivirus, dual-fluorescence-positive cells were sorted by fluorescence-activated cell sorting (FACS) and then used to determine the off-target effects of mitoTALENs on nuclear genomes (Fig. S3E). We also generated TALENs with a nuclear localization signal (NLS) as controls, which target the 3243G sequence and the EGFP gene. To enrich the transfected cells, MitoTALENs or NLS-TALENs were co-electroporated with a *Puro* expression plasmid into the dual-fluorescence reporter cells. After selection with puromycin (0.5 µg/mL) for 2 days, FACS was performed to analyze the expression levels of the dual fluorescence markers, which showed that NLS-TALENs were highly efficient in targeting nuclear sequences and disrupted the expression of EGFP in 13%–20% of the transfected cells. In contrast, MitoTALENs targeted to the same sequence demonstrated a limited targeting ability for nuclear sequences, with only 3%–6% of the transfected cells shown to be mCherry^+^/EGFP^−^ (Figs. 3F and S3E).

### Metabolic rescue in patient-derived iPSCs by mitoTALENs

**Figure 4:**
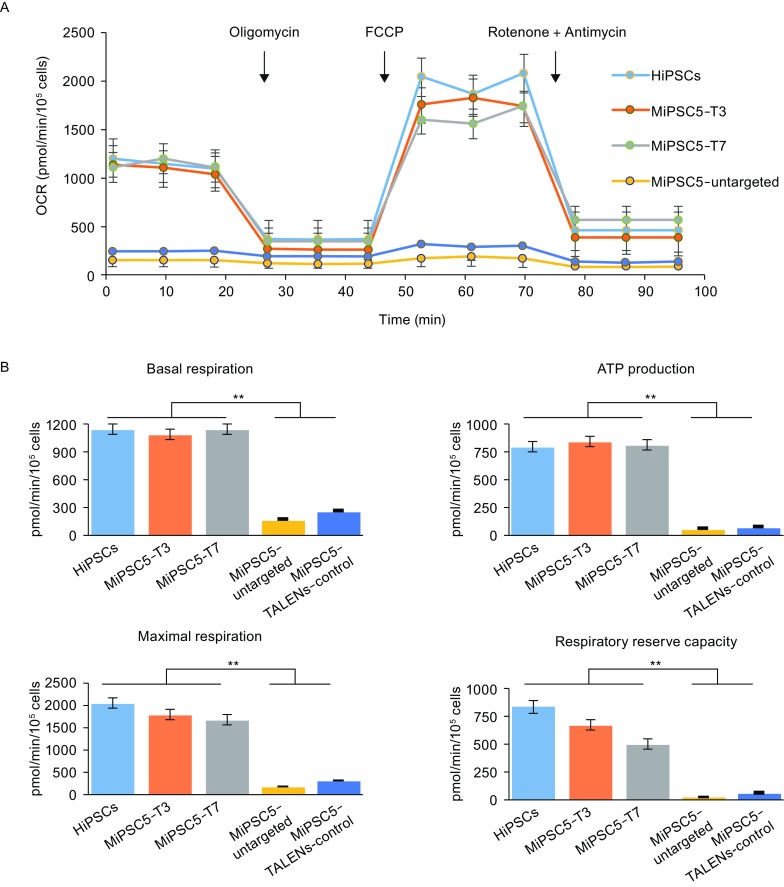
**Mitochondrial respiratory function of MELAS-iPSCs and targeted subclones**. (A) Mitochondrial function based on *in vitro* oxygen capacity in response to 0.5 µg/mL oligomycin, 1 µmol/L 4-(trifluoromethoxy) phenylhydrazone (FCCP), 0.5 µmol/L rotenone and 1 µmol/L antimycin. (B) Quantitative analysis of basal oxygen consumption, ATP production, maximal respiration and proton leak of iPSCs (*n* = 3, error bars represent ±SEM; **P < 0.05)

The A to G substitution at mtDNA nucleotide position 3,243 causes 80% of mitochondrial encephalomyopathy, lactic acidosis, and stroke-like episodes (MELAS), which affects many of the body’s systems, particularly the nervous system and the muscles (Goto et al., 1990). The 3243A>G mtDNA mutation disturbs the function of tRNA leucine 1 (UUA/G) and impairs the ability of mitochondria to make proteins, use oxygen, and produce energy. To evaluate the mitochondrial function of MiPSCs and to determine the genetic rescue of the sub-clones by mitoTALENs, oxygen consumption rates (OCRs) were determined using XF24 extracellular flux analyzers (Seahorse Biosciences), which indicated the mitochondrial respiration and energy production capacities. Compounds (oligomycin, FCCP, and a mix of rotenone and antimycin A) were serially injected to measure ATP production, maximal respiration, and non-mitochondrial respiration, respectively (Fig. 4A). MiPSCs harboring high 3243A>G heteroplasmy levels demonstrated significantly reduced OCRs compared with hiPSCs derived from a healthy person (Fig. 4A and 4B), while MiPSC sub-clones (MiPSC5-T3 and T7) genetically rescued by mitoTALENs exhibited functional recovery of mitochondrial respiration.

### Mitochondrial function in corrected MiPSC-derived NPC

**Figure 5:**
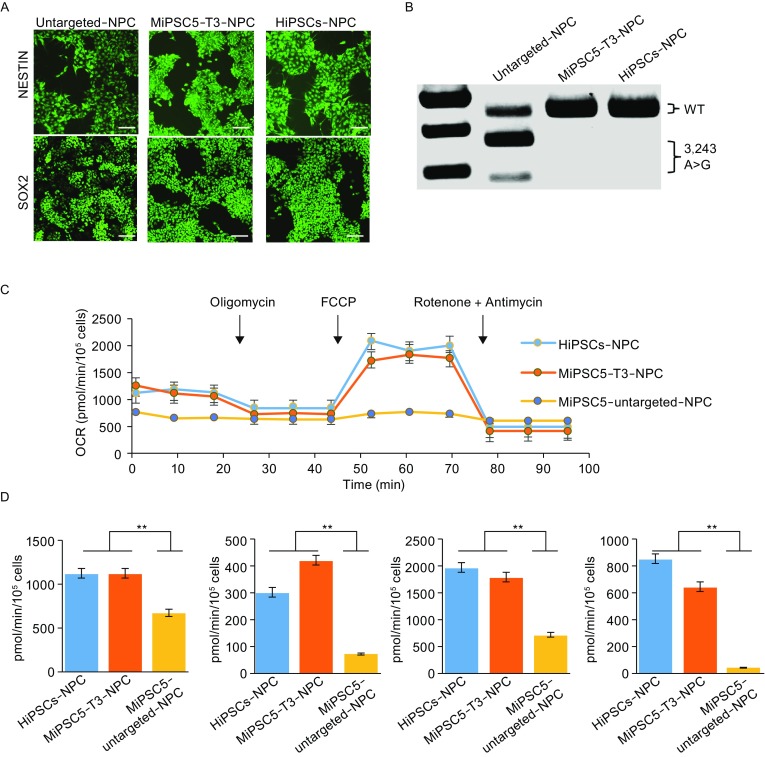
**Mitochondrial respiratory function of neural progenitor cells differentiated from MELAS-iPSCs**. (A) Immunofluorescence analysis of neural progenitor markers in MELAS-iPSC-derived NPCs. Scale bar, 100 mm. (B) RFLP analysis and quantification of m.3243A>G heteroplasmy in MELAS-iPSC-derived NPCs. (C) OCR of the NPCs differentiated from wild-type hiPSCs, MELAS-iPSCs and targeted subclones. (D) Quantitative analysis of basal oxygen consumption, ATP production, maximal respiration and proton leak of NPCs (*n* = 3, error bars represent ±SEM; **P < 0.05)

The distinguishing clinical feature of MELAS syndrome is the recurrence of stroke-like episodes. To further evaluate whether genetic targeting of mutant mtDNA could restore the mitochondrial function of neural progenitor cells (NPCs), NPCs were differentiated from WT hiPSC, MiPSCs and the targeted sub-clone MiPSC5-T3. Immunofluorescence staining was performed to confirm neural differentiation of iPSCs with the neural progenitor markers Nestin and Sox2 (Fig. 5A). The mutation rate of mtDNA 3243A>G in the NPCs was detected by RFLP (Fig. 5B) and showed no apparent differences with the iPSCs. Next, mitochondrial functions of the NPCs were investigated using XF24 extracellular flux analyzers (Fig. 5C and 5D). OCR data indicated that mitochondrial function of the iPSC-derived NPCs was successfully restored after gene correction.

### Specific reduction of human mutant mtDNA in porcine oocytes

**Figure 6:**
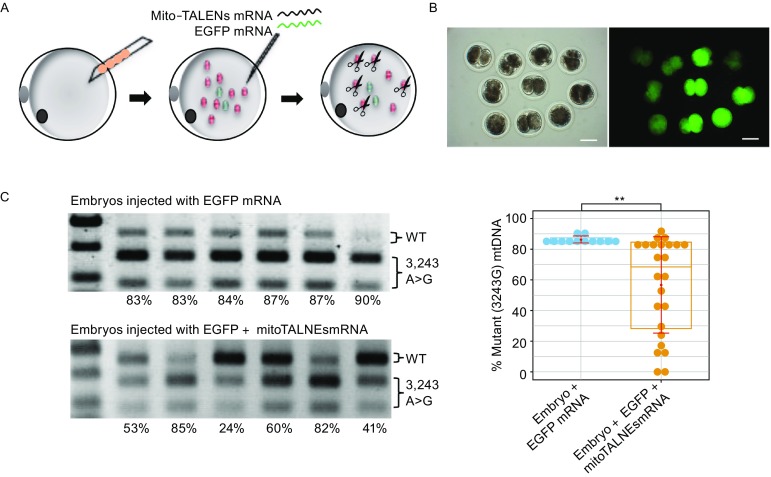
**Specific targeting of human mutant mtDNA in porcine oocytes using MitoTALENs**. (A) Construction of porcine oocytes carrying human m.3243G>A mutations by injection of the cytoplasm of MiPSCs into porcine MII oocytes, followed by injection of EGFP and mitoTALENs mRNA targeting the 3243G mutant mtDNA. (B) Expression of EGFP in artificial porcine oocytes 48 h after injection of mRNA. (C) RFLP analysis and quantification of m.3243A>G heteroplasmy in individual oocytes 3 days after mRNA injection (EGFP *n* = 12; EGFP + TALENs *n* = 24, error bars represent ±SEM; **P < 0.05)

To evaluate the specific elimination of mutant mtDNA in patient-derived iPSCs and the potential use of mitoTALENs to prevent the germline transmission of mutant mtDNA, we analyzed the ability of mitoTALENs to reduce mutant mtDNA levels in mammalian oocytes. A previous study showed that mitochondria-targeted nucleases could prevent germline transmission of mutant mtDNA in mice (Bacman et al., 2010). Several other mitoTALENs have also successfully reduced human m.14459G>A and m.9176T>C mutant mtDNA in murine oocytes (Reddy et al., 2015). Using porcine oocytes, which have a similar developmental timeline as human oocytes (Griffin et al., 2006), we generated artificial porcine oocytes carrying the human m.3423A>G mtDNA mutation by direct injection of the cytoplasm of MiPSCs into porcine MII oocytes (Fig. 6A). *In vitro* transcribed mitoTALENs mRNA was then injected into the oocytes harboring human m.3423A>G mtDNA. To monitor gene expression, EGFP mRNA was co-injected into the oocytes. The expression of EGFP was assessed by fluorescence microscopy after 48 h (Fig. 6B), after which RFLP analysis was performed to detect the levels of 3243A>G heteroplasmy. Compared with the control (where only EGFP mRNA was injected), the injection of mitoTALEN mRNA significantly reduced the human 3243A>G mutant mtDNA (Figs. 6C and S4**)**. Collectively, these results demonstrated the potential for custom-designed mitoTALENs to specifically eliminate disease-relevant mtDNA mutations responsible for human mitochondrial diseases.

## Discussion

Mitochondrial diseases refer to a group of clinically heterogeneous disorders caused by mitochondrial dysfunction. Because mitochondria are not only regulated by the nuclear genome but also by their own mtDNA, mutations within either set of DNA may cause mitochondrial disorders. Unlike the nuclear genome, mutations in mtDNA occur at a higher rate (Ingman et al., 2000). However, there is no proven technology that allows the generation of targeted mtDNA mutations to study the basic characteristics of mitochondrial biogenesis. This has prompted keen interest in the development of relevant cellular and animal models and in exploring innovative therapeutic strategies to modulate the mitochondrial deficiencies observed in these diseases. In this study, we have focused on genetically correcting mitochondrial disease patient iPSCs by targeting the disruption of mutant mtDNA using mitochondrial-targeted TALENs. MiPSCs harboring a high ratio of the pathogenic mtDNA mutation m.3243A>G were generated from a MELAS patient. We first engineered mitoTALENs, which exhibited a highly specific mutant mtDNA targeting capability. Through the transient expression of mitoTALENs, we achieved a remarkable reduction in mutant mtDNA and demonstrated the functional metabolic rescue of MiPSCs and derived NPCs. Moreover, the injection of mitoTALEN mRNA led to a significant reduction in human mutant mtDNA in reconstructed porcine oocytes.

Induced pluripotent stem cells (iPSCs) (Takahashi et al., 2007) derived from patients with genetic disorders hold enormous promise for basic research and drug screening. Specifically, mitochondrial disease-specific iPSCs carrying various heteroplasmic mtDNA mutations have been reported, which has opened new avenues for understanding the definitive genotype-phenotype relationship of affected tissues and organs in various types of mitochondrial diseases triggered by mtDNA mutations (Hatakeyama and Goto, 2016). iPSC lines harboring the m.3243A>G mtDNA mutation were generated from patient fibroblasts with various levels of heteroplasmy. In addition, isogenic iPSCs carrying exclusively wild-type or mutant mtDNA were reported and were generated though the spontaneous segregation of heteroplasmic mtDNA during reprogramming or proliferation of patient fibroblast (Ma et al., 2015). Although we did not obtain these iPSCs in this experiment, this might be because the fibroblasts derived from the patient carried a ratio of m.3243A>G mtDNA mutation above the pathogenic threshold level, which limited the proliferative ability of the cells, resulting in the failure of segregation of the heteroplasmic mtDNA. As reported, we also found mtDNA mutations that did not affect the reprogramming of the fibroblasts or the stability and pluripotency of iPSCs (Hamalainen et al., 2013). This might have been because the iPSCs predominantly produced energy via anaerobic glycolysis (Ma et al., 2015).

Due to the lack of a DSB repair pathway, DSBs caused by various effectors will lead to degradation of damaged mtDNA (Moretton et al., 2017). Engineered mitochondria-targeted nucleases, including restriction endonucleases and customized endonucleases, have been used to specifically eliminate mutant mtDNA in human tumor-derived cell lines and murine models. Compared with restriction endonucleases and ZFN, TALENs can be more flexibly designed and constructed using a simple “one-to-one” correspondence between single DNA base pairs in a target site and two-amino-acid sequences in one TAL effector repeat (Boch and Bonas, 2010). Although CRISPR/Cas9 technology has been widely used in targeting nuclear DNA, only one recent study showed that mtDNA editing is possible using CRISPR/Cas9 (Jo et al., 2015). Additionally, unlike other endonucleases, the specific DNA targeting of Cas9 was guided by an engineered guide-RNA by means of base-pair complementarity. Thus, reengineered guide-RNA would require the development of mitochondrial localization- and single-base-pair-distinguishing capabilities. While our current study and those previously reported have demonstrated that engineered mitoTALENs can specifically target single-base pair mutant mtDNA, we have utilized a dual fluorescence reporter to illustrate the limited targeting ability of mitoTALENs to the nuclear genome.

Interestingly, using Illumina MiSeq to sequence complete mtDNA, two heteroplasmic variants were found in rescued MiPSC sub-clones that were different from the untargeted MiPSCs. As the current study suggested that the mtDNA mutation frequency is significantly increased in iPSCs, the additional variants of rescued MiPSCs observed in this study might have developed during the *in vitro* culturing and editing. Another possibility is that the nontargeted MiPSCs also included those variants at a rare frequency, but their frequency accumulated in the mito-TALEN-induced mtDNA heteroplasmy shifts. Regardless, this implied that comprehensive assessment of variants in mtDNA is necessary when using engineered nucleases to genetically correct mitochondrial diseases.

In contrast to the nuclear genome, mtDNA is segregated in a non-Mendelian manner and is exclusively transmitted through maternal inheritance (Ingman et al., 2000). Therefore, MRT has been developed as a therapeutic approach to prevent germline transmission of mutant mtDNA and has been approved in the UK as well as declared ethically permissible in the US. Despite MRT showing great potential, there are serious ethical concerns surrounding this approach, and more studies are required to show the effects of MRT in human embryonic development and reproduction (Yamada et al., 2016). Our study, together with a previous study, highlights the significant potential of mitoTALENs for the specific elimination or correction of disease-relevant mtDNA mutations that are responsible for mitochondrial diseases in humans. Furthermore, the approach used to reconstruct porcine oocytes via injection of the cytoplasm of iPSCs combined with mitoTALEN mRNA serves as a proof-of-concept for the successful prevention of transmission of mutant mtDNA as well as therapeutic compensation for the reduced mitochondrial copies.

## Materials and methods

### Animals and ethics statement

The patients in this study provided written informed consent prior to donating fibroblasts for stem cell generation. The experiments regarding animal research were approved by the Institutional Review Board at The Third Affiliated Hospital of Guangzhou Medical University. The experiments using human cells and mice were approved by the ethics committee of The Third Affiliated Hospital of Guangzhou Medical University, and all animal care and experiments were performed in accordance with the institutional ethical guidelines for animal experiments.

### MELAS patient iPSC generation

A skin biopsy was obtained from a MELAS patient. Human fibroblasts were cultured in fibroblast medium (DMEM) supplemented with 10% fetal bovine serum (FBS) (HyClone), 1 mmol/L glutamine (Gibco), 1% non-essential amino acids (NEAA) (Gibco), and 100 IU/mL penicillin/streptomycin (Gibco). To generate iPSCs, fibroblast cells were transfected with a Sendai virus reprogramming kit (Life Technologies, A16517). The transfected cells were then plated onto Matrigel-coated culture dishes according to the manufacturer’s instructions. To remove the Sendai viruses, iPSCs were incubated at 38.5°C for 4 days as instructed. All iPSCs were cultured on Matrigel-coated tissue culture dishes (ES-qualified, BD Biosciences) with mTeSR1 (STEMCELL Technologies) at 37°C and 5% CO_2_ in a 100% humidified atmosphere incubator. The culture medium was changed daily until the cells were ready for passage or harvest. The cells were passaged every 3–4 days using Accutase (Stemcell Technologies).

### MitoTALEN construction and activity determination

TALENs targeting the mtDNA 3423 locus were constructed through Golden Gate TALEN Assembly. TALEN expression vectors were pCAG-T7-TALEN (Sangamo)-Destination with heterodimeric (ELD, KKR) domains obtained from Addgene (Plasmids #37184 and #40131).

A single strand annealing (SSA) assay was performed to detect the activity of TALENs as previously reported. TALEN-targeted sequences, both mtDNA 3243A and 3243G, were amplified from MiPSC and HiPSC genomes using the following primers with additional *Bam*HI and *Hin*dIII enzyme sites: forward, 5′gcgcggatccgagaaataaggcctacttca3′; reverse, 5′gcccaagcttatgccattgcgattagaatg 3′. The PCR fragment digested with *Bam*HI and *Hin*dIII was inserted into a pSSA-EGFP vector, and the correct clones were confirmed by Sanger sequencing. For SSA assays, 0.5 μg TALEN expression plasmid and SSA reporter were co-electroporated into 5.0 × 10^5^ HEK293 cells using a Neon transfection system according to the manufacturer’s instructions (Life Technologies). The expression of the EGFP was observed under a fluorescence microscope using appropriate filters after 48 h, and the proportion of the EGFP-positive cells was measured by flow cytometry.

To construct the mitoTALENs, the MTS coding sequences were first synthesized and cloned into the pEF1α-puro vector by replacement of the *puro* cassette, designated pEF1α-MTS. The sequences used for encoding MTS are listed below:

- APE1-MTS:
- ATGCACTCTCTGTTACCTGCATTGTGTGACAGCAAGATCCGTTCCAAGGCCCTCGGCAGTGATCACTGTCCTATCACCCTATACCTAGCACTG
- ATP5B-MTS:
- ATGTTGGGGTTTGTGGGTCGGGTGGCCGCTGCTCCGGCCTCCGGGGCCTTGCGGAGACTCACCCCTTCAGCGTCGCTGCCCCCAGCTCAGCTCTTACTGCGGGCCGCTCCGACGGCGGTCCATCCTGTCAGGGACTATGCGGCGCAAACATCTCCTTCG
- COX8A–MTS:
- ATGTCCGTCCTGACGCCGCTGCTGCTGCGGGGCTTGACAGGCTCGGCCCGGCGGCTCCCAGTGCCGCGCGCCAAGATCCATTCGTTG
- COX10-MTS:
- ATGGCCGCATCTCCGCACACTCTCTCCTCACGCCTCCTGACAGGTTGCGTAGGAGGCTCTGTCTGGTATCTTGAAGTCGACGCG
- SOD2-MTS:
- ATGTTGAGCCGGGCAGTGTGCGGCACCAGCAGGCAGCTGGCTCCGGTTTTGGGGTATCTGGGCTCCAGGCAGAAGCACAGCCTCCCCGACCGCGTCGACCGC

The mitoTALENs targeted to the 3243G mtDNA were constructed by subcloning the TALEN monomers from pCAG-T7-TALENs into pEF1α-MTS and then removing the nuclear localization signal. We generated the mitoEGFP vectors by cloning an EGFP cassette without the start codon ATG into the pEF1α-MTS vectors. Meanwhile, mitoTALEN-EGFP was constructed by one-step subcloning of the TALEN-LWT monomer and EGFP into pEF1α-MTS using a Gibson Assembly kit from New England Biolabs.

### Mitochondrial genome targeting by mitoTALENs

Approximately 1 × 10^6^ MiPSCs were electroporated using a Neon transfection system (Life Technologies) at 1,150 V, 30 ms, and 1 pulse in 100 µL of buffer B containing 4 μg of each TALEN monomer and 2 µg pEGFP-N1(Clontech) as a selection maker. The cells were then recovered in mTeSR1 medium supplemented with 10 mmol/L Rho-associated kinase (ROCK) inhibitor Y-27632 (10 mmol/L, Sigma) after electroporation. At 48 h post-transfection, the GFP-positive cells were collected via FACS and plated in 6-well plates. After 10–14 days of culture, single colonies were picked and cultured in 96-well plates for further expansion and identification.

### Heteroplasmy determination by RFLP

Total DNA from cells was extracted using the TIANamp genomic DNA kit (Tiangen). For RFLP analysis, the mtDNA 3243 locus was amplified using the following primers: forward, 5′cctcggagcagaacccaacct3′; reverse, 5′cgaagggttgtagtagcccgt3′, which produced a PCR product of 634 bp. In the presence of the A3243G mutation, the PCR product was digested by ApaI into two fragments of 424 and 210 bp. The digested PCR products were separated on a 1% agarose gel and stained with ethidium bromide. ImageJ software (https://imagej.nih.gov/ij/) was used to analyze the signal intensity of the bands.

### Neural progenitor cell differentiation and culture

A STEMdiffTM Neural System (Stemcell technologies) was used to differentiate neural progenitor cells from iPSCs. iPSCs were harvested by treatment with 2 mg/mL dispase (Invitrogen) and then washed twice with DPBS. The cells were resuspended in STEMdiffTM Neural Induction Medium with 10 μmol/L Y-27632 and plated onto Matrigel-coated culture dishes at an approximate density of 2 × 10^5^ cells/cm^2^. A daily full medium change was performed with warm STEMdiffTM Neural Induction Medium. Cells were passaged three times upon reaching 70% to 80% confluence using Accutase. Then, NPCs were maintained on Matrigel-coated dishes in STEMdiffTM Neural Progenitor Medium.

### Oxygen consumption detection

To assess the mitochondrial respiration and energy production of the MiPSCs, oxygen consumption rates (OCR) were measured using XF24 extracellular flux analyzers (Seahorse Biosciences). Briefly, 5 × 10^4^ iPSCs were plated into the wells of a Matrigel-coated XF24 cell culture microplate and incubated with 10 μmol/L Y-27632 for 24 h to ensure attachment. Before the assay, the cells were equilibrated in unbuffered XF assay medium supplemented with 25 mmol/L glucose, 1 mmol/L sodium pyruvate, 2 mmol/L Glutamax, 1× nonessential amino acids and 1% FBS in a non-CO_2_ incubator for 1 h. The mitochondrial processes were interrogated by sequential injection of oligomycin (0.5 mg/mL), carbonyl cyanide 4-(trifluoromethoxy) phenylhydrazone (FCCP, 1 mmol/L) and rotenone (0.5 mmol/L)/antimycin A (1 mmol/L). The results were normalized to the cell number and analyzed using Seahorse XF24 software.

### Whole mtDNA sequencing

Mitochondrial whole-genome sequencing was performed using new next-generation sequencing (NGS) technology, VariantProTM Capture Technology (VPCT), designed by LC Sciences. Briefly, the sequence libraries were prepared by multiplex PCR using VariantPro primer pools, which contained 110 primer pairs in two PCR tubes with an average amplicon length of 200 bp. The amplicons were purified with Agen-court AMPure XP beads (Beckman Coulter Genomics, High Wycombe, UK) mixed in equimolar concentrations, and mitochondrial genome sequencing was performed on an Illumina HiSeq 4000. Globally, approximately 89.12% of the reads mapped to the reference sequence. The average counts per amplicon were over 1000. Mitochondrial heteroplasmy rates in each sample were calculated as the percentage of the number of alternative reads to that of the total reads. The clinical significance of the variants was then analyzed with MitoMaster (http:// www.mitomap.org/MITOMASTER/WebHome).

### Immunofluorescence staining and fluorescence analysis

For pluripotent analysis, iPSCs were fixed with 3% paraformaldehyde for 15 min, permeabilized with 0.1% Triton-X and blocked using Super-block. Immunofluorescence (IF) staining was performed using primary antibodies (all at 1:200 dilutions) to detect OCT4 (Abcam), SOX2 (Abcam), TRA-1-60 (Abcam), and SSEA-4 (Abcam). The nuclei were stained with DAPI at a final concentration of 0.01 mg/mL for 10 min.

For the mitochondrial localization analysis, mitoEGFP- and mitoTALEN-EGFP-transfected cells were incubated in the presence of 200 nmol/L MitoTracker Red CMXRos and 0.5 µg/mL Hoechst (Invitrogen) at 37°C for 30 min and fixed with 1% paraformaldehyde in PBS for 10 min. Confocal image acquisition was performed using a Zeiss LSM 710 laser-scanning microscope (Carl Zeiss Jena). Line profiles were performed on images using the RGB Profiles Tool in ImageJ.

### Reconstruction of porcine oocytes and mRNA injection

Porcine ovaries were obtained from a local slaughterhouse. Oocytes were aspirated from 3 to 6 mm follicles and cultured in TCM-199 supplemented with 0.1% polyvinyl alcohol, d-glucose (3.05 mmol/L), sodium pyruvate (0.91 mmol/L), penicillin (75 µg/mL), streptomycin (50 µg/mL), epidermal growth factor (10 ng/mL), cysteine (0.57 mmol/L), follicle-stimulating hormone (0.5 µg/mL), and luteinizing hormone (0.5 µg/mL) at 38.5°C at 5% CO_2_ for 42 to 44 h. Oocytes with an extruded first polar body, a round shape and an intact cytoplasm were selected and maintained in the manipulation medium for subsequent experiments. Oocyte activation was performed in medium containing 0.3 mol/L mannitol, 1.0 mmol/L CaCl_2_·2H_2_O, 1.0 mmol/L MgCl_2_·6H_2_O and 0.5 mmol/L HEPES, using two 2-DC pulses with a voltage of 1.2 kV/cm for 30 µs on a BTX Electro Cell Manipulator 2001 (BTX, San Diego, CA, USA). Oocytes were then cultured in PZM-3 (with 3 mg/mL BSA) supplemented with 7.5 µg/mL cytochalasin B for 4 h. The cytoplasm of the MiPSCs was extracted by gentle aspiration of cells in and out of the injection pipette. Cytoplasm from 4–5 cells was injected inside the ooplasm using the micropipette. After 3–4 h of recovery in PZM-3 (with 3 mg/mL BSA), reconstructed oocytes were injected with mitoTALENs (200 ng/µL) and EGFP (50 ng/µL) mRNA and then further cultured in PZM-3 (with 3 mg/mL BSA) at 38.5°C in a humidified atmosphere at 5% CO_2_ for 3 days before analysis. The mRNA was produced using a mMESSAGE mMACHINE SP6 ULTRA kit (Life Technologies) according to the manufacturer’s instructions using linearized and gel-purified (QIAGEN) plasmid templates.

### Teratoma formation and karyotype analysis

Approximately 1–2 × 10^6^ iPSCs from a confluent 10-cm plate were harvested by digestion with 2 mg/mL dispase, resuspended in Matrigel, and injected into the inguinal grooves of 6-week-old male SCID mice. Eight weeks later, the resulting tumors were removed, fixed for 4–8 h in 4% paraformaldehyde, and embedded in paraffin. After staining with hematoxylin and eosin (H&E), the sections were examined using a light microscope to determine the presence of derivatives from the three germ layers.

For the chromosome analysis, iPSCs were incubated in culture medium with 0.25 mg/mL colcemid (Gibco, Invitrogen) for 4 h, harvested, and incubated in 0.4% sodium citrate and 0.4% chloratum Kaliumat (1:1, *v*/*v*) at 37°C for 5 min. The cells were then fixed three times in a methanol:acetic acid solution (3:1, *v*/*v*). Subsequently, after Giemsa staining, at least 20 cells were examined in each group for the chromosome analysis.

### Short tandem repeat analysis (STR)

For STR analysis, the genomic DNA was extracted from MiPSCs, targeted MiPSCs and the patient’s fibroblasts. The extracted DNA was amplified for 15 different genetic loci using a Promega PowerPlex 16 system kit (Promega). Capillary electrophoresis was performed on an automated ABI 3100 genetic analyzer (Applied Biosystems).

### Statistical analysis

All statistical analyses were performed using SPSS 19.0 software to detect significant differences in the measured variables among the groups. A value of *P* < 0.05 was considered to indicate a statistically significant difference.

## Electronic supplementary material

Below is the link to the electronic supplementary material.

Supplementary material 1 (PDF 638 kb)

Supplementary material 2 (XLSX 16 kb)
