# Supplementary material for: Targeted elimination of mutant mitochondrial DNA in MELAS-iPSCs by mitoTALENs
Source: Protein Cell. 2018 Jan 9;9(3):283–97. doi: 10.1007/s13238-017-0499-y (PMC5829275; doi:10.1007/s13238-017-0499-y)
Supplement: Supplementary file 1 — Supplementary material 1 (PDF 638 kb) [file 13238_2017_499_MOESM1_ESM.pdf]

Supplementary figure legends

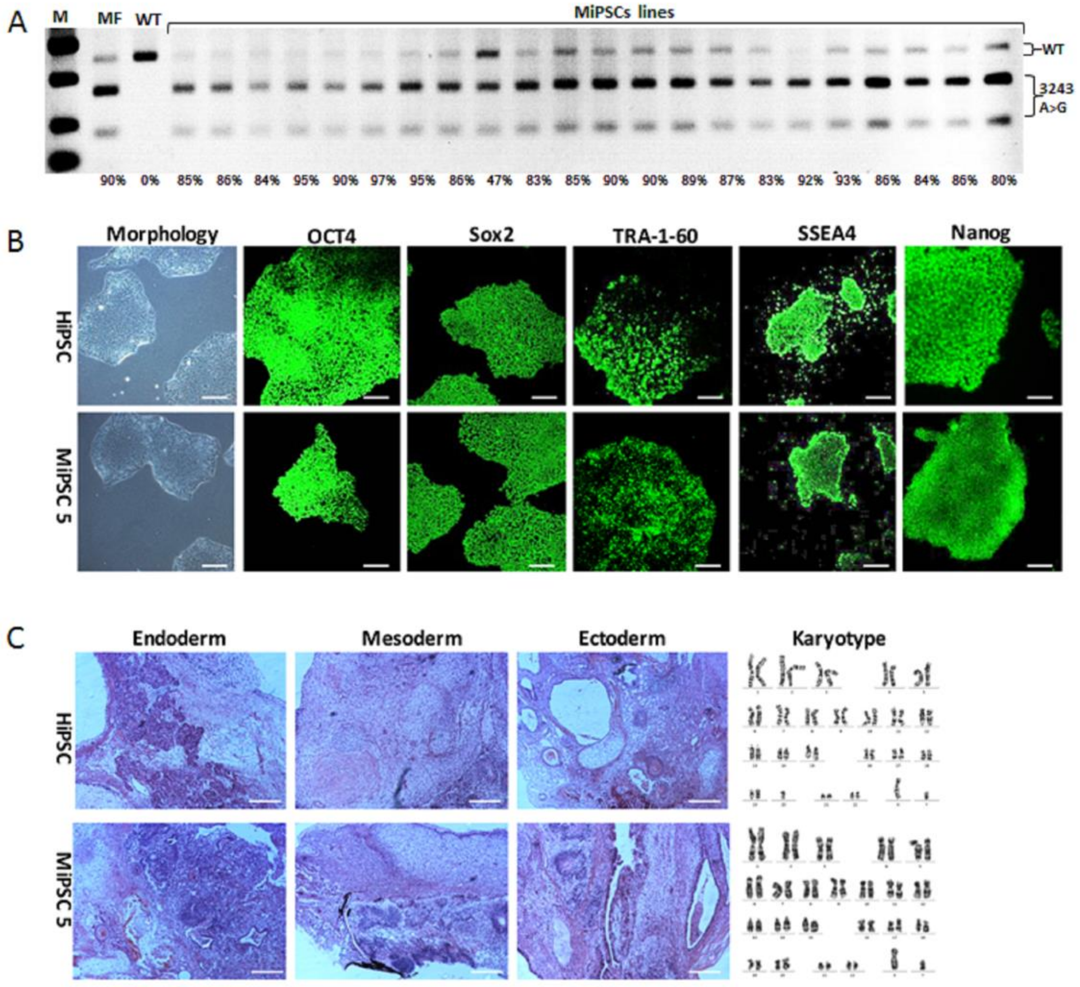

Supplementary figure 1 Establishment of iPSCs from MELAS patient somatic cells

A. MELAS patient-specific iPSCs (MiPSCs) lines were established which harbored high 3243A>G heteroplasmy levels (>80%). The mtDNA 3243A>G mutation rate was detected by RFLP analysis. MF: MELAS fibroblasts, WT: iPSC line (HiPSCs) derived from health human fibroblasts.

B. Immunofluorescence analysis revealing the expression of pluripotency markers (OCT4, SOX2, TRA1-60, SSEA4 and NANOG) in the MiPSC5 subline at passage 30, which still harbored high 3243A>G heteroplasmy levels (87%). The HiPSC line was used as a control.

C. Sections of the teratomas derived from MiPSC5 and HiPSCs showing the differentiation of histologically distinct tissues, including gut (endoderm), cartilage (mesoderm), and adipose (mesoderm). Normal karyotype of a representative MiPSC5. Scale bar = 200 μm.

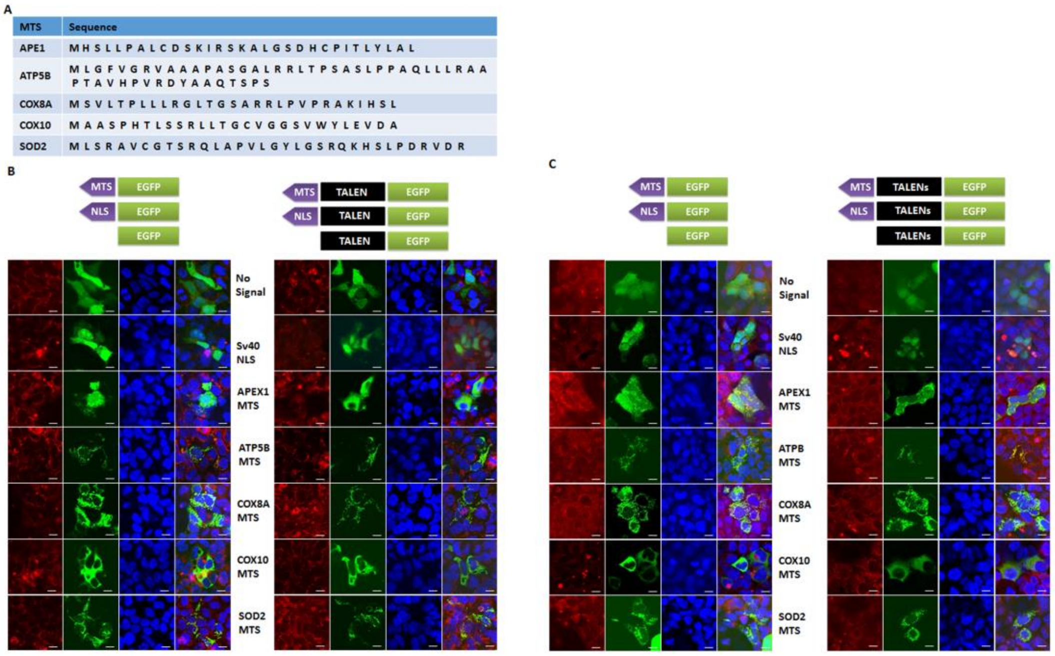

Supplementary figure 2

Fluorescence colocalization analysis of mitochondrial localization of EGFP and TALEN-EGFP driven by endogenous MTSs

A. Amino acid sequence of MTSs derived from nuclear genesAPEX1, ATP5B, COX8A, COX10, and SOD2

B, C. Mitochondrial co-localization of EGFP and TALENs monomers in human iPSCs(B) and HEK293 cell line (C) with Mitotracker. Scale bars, 10 mm.

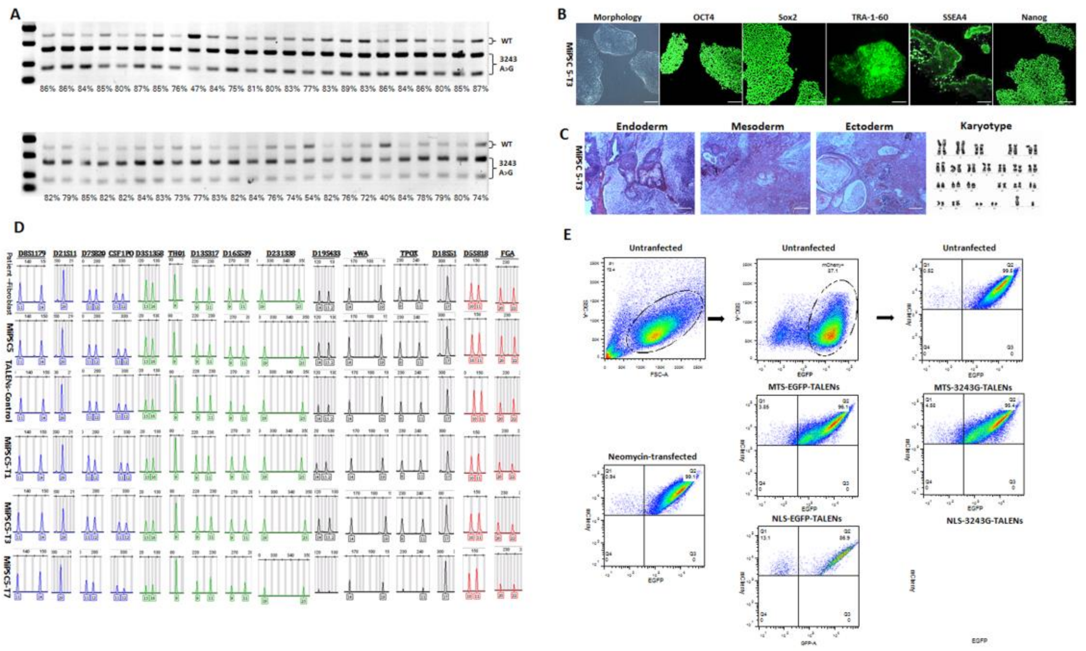

Supplementary figure 3

Characterization of genetically rescued MiPSCs.

A. RFLP analysis and quantification of mtDNA 3243A>G heteroplasmy in untargeted and TALENs control MELAS iPSCs.

B. Immunofluorescence analysis the expression of pluripotency markers (OCT4, SOX2, TRA1-60, SSEA4 and NANOG) in mitoTALENs targeted MiPSC5 subclone3 , which was homoplasmic for the wild-type allele.

C. Sections of teratomas derived from targeted MiPSC5 subclone 3 showing the differentiation of histologically distinct tissues, including gut (endoderm), cartilage (mesoderm), adipose (mesoderm). Normal karyotype of representative MiPSC5-T2. Scale bar = 200µm.

D. Fingerprinting by short tandem repeat analysis (STR) the MiPSCs primary lines and targeted clones within 15 STR loci.

E. Dual fluorescent iPSC reporter analysis the capability of mitoTALENs targeting on nuclear genome.

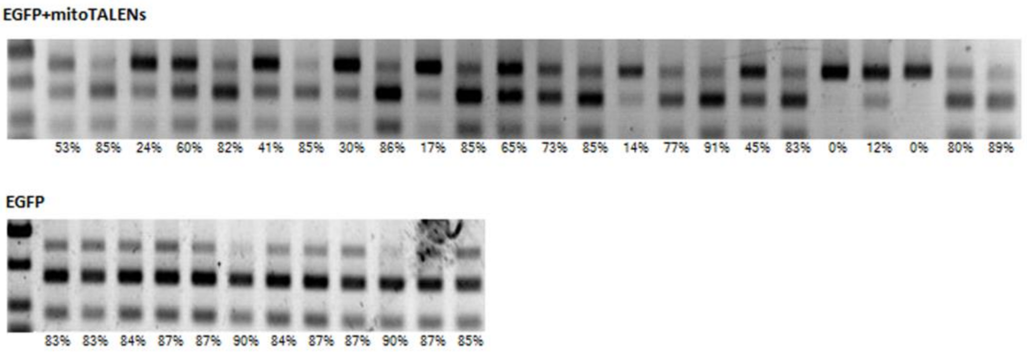

Supplementary figure 4

Specific targeting of human mutant mtDNA in porcine oocytes via injection of mitoTALENs mRNA .

RFLP analysis and quantification of m.3243A>G heteroplasmy in individual oocytes with mRNA injection after 3 days

Supplementary Table 2 3243G mito-TALENs off-targets

Chr	Location	Gene	obs.size	exp.size	L m/g	R m/g
1	238,136,891.. …238,137,024 (-)	-	134 >	32-100	2-Jan	2-Jan
	Targets<br><br>Genome	GCGATTACCGGGCCCT<br><br>GC-ATTACTGGCCCCT	<R<br><br>...	L><br><br>...	TGTTCTTGGGTGGGTG<br><br>TGTTCTTTGG-GGCTG	
2	123,665,295.. …123,665,362 (+)	-	68	32-100	1-Feb	2-Feb
	Targets<br><br>Genome	C-ACCCACCCAAGAACA<br><br>CGACCC-CCCAAGAAGA	<L<br><br>...	R><br><br>...	AGGGCCCGGTAATCGC<br><br>ATGGCCCAGTAA-C-C	
